# Star-Like Microgels vs Star Polymers: Similarities and Differences

**DOI:** 10.1021/acs.macromol.6c00287

**Published:** 2026-05-05

**Authors:** Tommaso Papetti, Elisa Ballin, Francesco Brasili, Emanuela Zaccarelli

**Affiliations:** † Department of Physics, 9311 Sapienza University of Rome, Piazzale Aldo Moro 2, Roma 00185, Italy; ‡ Uos Sapienza, 9327 CNR Institute of Complex Systems, Piazzale Aldo Moro 2, Roma 00185, Italy

## Abstract

Star-like microgels have recently emerged as a promising class of thermoresponsive soft colloids that have an internal architecture similar to that of star polymers. Here, we perform extensive monomer-resolved simulations to theoretically establish this analogy. First, we characterize the effective potential between star-like microgels, finding that it is Gaussian for an extended range of distances, in stark contrast to the Hertzian-like potential of standard microgels, but almost identical to that of star polymers with a core partially covered by chains. Next, we investigate the ratio between gyration and hydrodynamic radii across the volume-phase transition, showing qualitative agreement with both star polymers and experimental data. Finally, we estimate the bulk modulus, finding star-like microgels significantly softer than standard microgels and comparable to star polymers. The present work thus demonstrates that star-like microgels behave as ultrasoft particles, akin to star polymers, paving the way for their exploration at high concentrations.

## Graphical abstract

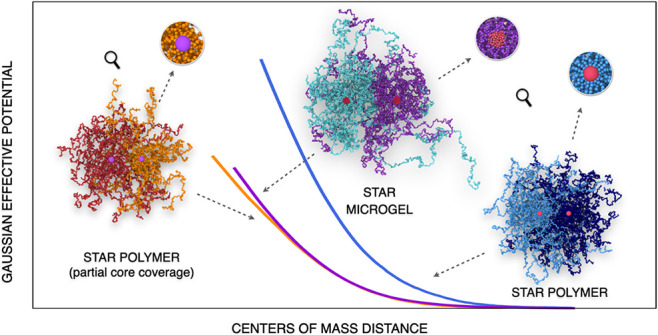

## Introduction

One of the key advantages to work with colloidal systems is their tunable *softness*, that can be modified by varying the internal architecture of the particles. A pioneering review article by Vlassopoulos and Cloitre^1^ has proposed to define softness as the ratio of the elastic free energy over the Boltzmann factor, *s* = *F*_el_/*k*_B_ *T*. This allows to classify colloidal particles from very soft, i.e., polymer chains with *s* ∼ *O*(1), to completely hard (hard spheres), with *s* → ∞. In between these extreme cases, there exists a variety of particles, often bridging together polymeric and colloidal nature, giving rise to a range of intermediate softness. Among these, we focus in this work on star polymers (*s* ∼ 10^3^) and microgels (*s* ∼ 10^4^).

Star polymers are colloidal particles made of long polymer chains, generally called arms, that are chemically attached to a common center, the core, whose dimension is much smaller than the arm length. Their behavior is essentially governed by the arm number *f* and has been extensively studied in the past both theoretically^2,3^ and experimentally.^4,5^ The classical theoretical description of star polymers is based on the well-established Daoud–Cotton blob model, where correlated blobs of increasing size depart from the central core. With this description, it was theoretically shown and numerically validated that the effective interaction potential between two stars, using as a natural variable the distance between the cores, increases logarithmically at short distances.^6,7^ Thanks to this very slow divergence at full contact, the potential between star polymers is often referred to as ultrasoft. At longer distances, there is not an analytic description of the potential, which was postulated to bend over a Yukawa form for *f*  > 10.^7^ At small enough *f*, a Gaussian description seems to be more accurate.^8^ These predictions were found to be in agreement with experiments^7^ and computer simulations^8^ for moderate values of arm numbers. However, star polymers can also be considered as deformable colloids and is possible to use the distance between their centers of mass as the variable to calculate their effective potential, similarly to what is commonly done with other macromolecules. In this case, it was shown by theoretical calculations and simulations on a lattice that the effective potential is not logarithmic, but rather adopts a Gaussian form^9^ which seems to hold in the whole range of relative distances.

A drawback acting against a wide use of star polymers in the experimental community is the rather involved synthetic process, which led in recent years to the search of alternative model systems with comparable properties. One notable example is given by asymmetric block copolymer micelles, assembling into star-like objects,^10^ whose phase behavior and interactions were found to be rather similar to that of ideal stars.^11,12^

On the other hand, microgels are cross-linked polymer networks, whose softness is normally controlled by the amount of cross-linkers *c*. Usually, they are synthesized by making use of thermoresponsive polymers, such as poly-*N*-isopropylacrylamide (PNIPAM), providing the microgels with the ability to respond to temperature. In particular, PNIPAM microgels are well-known to undergo a reversible Volume Phase Transition (VPT) at a temperature ∼32 °C, which makes them very suitable for a variety of applications.^13^ The standard preparation method is a simple precipitation polymerization, routinely employed by several groups around the world, which has favored their spreading in the community as a favorite model system for understanding the behavior of soft and deformable objects.^14,15^ Traditionally, bis­(acrylamide) (BIS) is the most used cross-linking agent. Since it reacts a bit faster than NIPAM, it yields the microgels with a characteristic internal structure, that is well-described by a fuzzy sphere model.^16^ This amounts to a denser core, rich in cross-linkers, and to a soft corona, poor of cross-linkers.

However, a recent work has put forward a new class of soft particles, which has the potential to bridge the gap between star polymers and microgels. These particles, named star-like microgels,^17^ are still thermoresponsive microgels based on PNIPAM, synthesized in an identical way to standard ones but with the only difference that the BIS cross-linker is substituted by another cross-linking agent: ethylene glycol dimethacrylate (EGDMA).^18^ The latter has a much higher reactivity than BIS, accumulating within a narrow central region of the microgels, analogous to the core of the star, and leaving the rest of NIPAM assembling into chains of various lengths, i.e., the arms. Reference 17 reported a joint experimental and numerical investigation of such microgels, aimed to characterize their internal structure under dilute conditions as a function of temperature across the VPT. The measured Small Angle X-ray Scattering (SAXS) form factors of the microgels were described with a hybrid model, combining star and microgel features, the so-called core-fuzzy sphere model, for two representative values of the EGDMA cross-linker concentration. For *c* = 1%, the microgels were found to retain star-like character, while for *c* = 10%, the core becomes much bigger and a simple star-like structure does not hold any longer. The data were then compared to monomer-resolved simulations of ideal star polymers, confirming a good agreement with ideal stars with *f* = 80 for *c* = 1%. To make a step forward, the *in silico* synthesis of realistic monomer-resolved microgels, put forward in refs 19 and 20 was also extended to describe the internal structure of PNIPAM-EGDMA microgels, finding remarkable agreement with experiments.

However, a fundamental question remains open, regarding the true similarity of star-like microgels with low EGDMA content to ideal star polymers. To answer this, in this work we exploit our *in silico* model to evaluate the effective interactions *V*_eff_ between star-like microgels with *c* = 1% and compare them with those of star polymers. Given that in the literature, numerical calculations of *V*_eff_ are not available for stars with large arm numbers, in the range needed to make a comparison with the present star-like microgels, we also provide new calculations of the star effective potentials at different arm numbers. On the other hand, effective interactions between standard microgels are usually reported to be Hertzian-like^21,22^ as typical of elastic spheres. We thus aim to shed light on the interactions between star-like microgels, as also compared to standard microgels and classify them among the variety of soft colloids.

Interestingly, we find that the calculated *V*_eff_ for star-like microgels is Gaussian, confirming their star-like character, but the resulting repulsion is significantly lower than for stars. We attribute this to the lower coverage of the core with respect to ideal stars achieved in PNIPAM-EGDMA microgels and thus compare our findings to a partially covered star, whose effective interactions are found almost identical to those of star-like microgels. In addition, we provide an estimate of the bulk modulus, finding that the star-like microgels are again similarly soft to star polymers, and much softer than regular microgels. In order to further validate the numerical model, we also report an estimate of the ratio between the gyration radius *R*_g_ and the hydrodynamic radius *R*_H_ as a function of temperature for all the simulated systems and compare them with new experimental measurements for star-like microgels, performed by the combination of static and dynamic light scattering.

The manuscript is organized as follows. After describing our numerical and experimental methods, we report the calculated effective potentials for star polymers, for star-like microgels, for regular microgels and for partially covered stars, comparing them to the known analytical and phenomenological forms and among themselves. We then also provide numerical estimates for *R*_g_/*R*_H_ for all these systems and compare them to literature for star polymers and to new measurements performed for star-like microgels. For the latter, we report the full temperature dependence in comparison to simulations, where this is varied by introducing a solvophobic attraction between the monomers. We then discuss our main findings in the context of existing literature, finding a strong analogy between star-like microgels and star polymers having a lower core surface coverage than ideal ones, as also confirmed by the analysis of the bulk modulus of the particles. Incidentally, we also show that this reduced coverage of the core surface does not qualitatively affect the shape of the effective interactions and the internal structure of the particles with respect to regular stars.

The present work thus theoretically establishes star-like microgels as a promising model system for investigating the role of softness in the behavior of dense colloidal systems, akin to star polymers and also able to respond to temperature.

## Numerical Methods

### Star Polymer Model

We construct a star polymer by placing a single central monomer with a finite radius *R*_c_ and decorating it with *f* arms, each made of *N*_*f*_ monomers of diameter σ and unit mass *m*. We vary *f* in the range from 6 to 80, the latter value being the estimate of the arm number for star-like microgels established in ref 17. For the latter case, we consider *N*_*f*_ = 200, but we also report selected results for *N*_*f*_ = 50. The effective coverage fraction, defined as $\gamma =f/16R_{c}^{2}$ , is set to 1,^2^ hence *R*_c_ ≃ 2.2σ. However, to compare with star-like microgels, we also perform simulations of nonideal stars, where we vary the value of γ. That is accomplished either by covering the core (with the same size as above) with a smaller number of arms, or by fixing the latter and changing the core radius in the range 1.7 σ ≲ *R*_c_ ≲ 4σ. In all cases we start with a configuration where arms are fully stretched, that is later equilibrated with the monomer interactions described below.

### Microgel Model

The assembly of the microgels is performed as in previous works.^19,20^ This is by now a well-established procedure, that exploits the assembly of bivalent patchy particles (the monomers) and tetravalent patchy particles (the cross-linkers), all with diameter σ in a spherical volume, with number density ρ ∼ 0.08, fixed by the favorable comparison with experiments.^20^ During the assembly, the cross-linkers experience an additional force, which phenomenologically represents their higher reactivity, which accumulates them preferentially in the center of the microgels. This force is found to be always the same for PNIPAM-BIS microgels with any *c* and was generalized in ref 17 to the case of PNIPAM-EGDMA microgels. In addition, EGDMA monomers can form bonds between themselves, while for BIS ones this possibility is usually neglected.

Using these protocols, here we assemble star-like microgels starting with *N*_m_ = 21000 particles, of which 1% is the molar amount of cross-linkers. For comparison, we also prepare *standard* (also sometimes called *regular*) microgels, having the same cross-linker concentration and a similar *N*_m_. These microgels have a standard fuzzy sphere architecture as those studied in our previous works.^20,23^ In all cases, when most bonds (>99.5% of the total) are formed, we select the largest cluster as the obtained microgel networks, whose topology is then fixed. The resulting microgels have *N*_m_ ∼ 20000 for regular microgels and *N*_m_ ∼ 16000 for star-like microgels.

### Interaction Potentials in Monomer-Resolved Models

For both the obtained star polymers and microgels, all pair interactions are modeled with the bead–spring model.^24^ More precisely, the steric repulsion is modeled via a Weeks–Chandler–Andersen (WCA) potential, acting between two beads *i*,*j* with diameters σ_*ij*_ = (σ_*i*_ + σ_*j*_)/2, thus also including the core of the star polymers:

1 $$V_{\mathrm{WCA}}\left(r\right)=\left\{ \begin{array}{ll} 4\epsilon \left[{\left(\frac{\sigma_{ij}}{r}\right)}^{12}-{\left(\frac{\sigma_{ij}}{r}\right)}^{6}\right]+\epsilon , & \mathrm{if}\;r\leq 2^{1/6}\sigma_{ij},\\ 0, & \mathrm{otherwise} \end{array}\right.$$

with ϵ the unit of energy and *r* the distance between two particles. In all simulations, the monomer diameter σ is the unit of length. Bonded particles additionally interact with the finitely extensible nonlinear elastic (FENE) potential

2 $$V_{\mathrm{FENE}}\left(r\right)=-\epsilon k_{F}R_{0}^{2}\;\ln\left[1-{\left(\frac{r}{R_{0}\sigma_{ij}}\right)}^{2}\right],\mathrm{if}\;r<R_{0}\sigma_{ij}$$

with bond stiffness set to *k*_F_ = 15 and maximum extension *R*_0_ = 1.5. The temperature-dependent quality of the solvent is mimicked by adding to the bead–spring model a solvophobic potential,^25^ routinely adopted for PNIPAM–BIS microgels:^20^

3 $$V_{\alpha }\left(r\right)=\left\{ \begin{array}{ll} -\epsilon \alpha , & \mathrm{if}\;r\leq 2^{1/6}\sigma_{ij},\\ \frac{1}{2}\alpha \epsilon \left[\cos\left(\gamma {\left(r/\sigma_{ij}\right)}^{2}+\beta \right)-1\right], & \mathrm{if}{\;2}^{1/6}\sigma_{ij}<r<R_{0}\sigma_{ij},\\ 0, & \mathrm{if}\;r>R_{0}\sigma_{ij} \end{array}\right.$$

with γ = π­(2.25 – 2^1/3^)^−1^ and β = 2π – 2.25γ. This attractive potential implicitly accounts for solvent effects and it is controlled by the solvophobic parameter α, which acts as an effective temperature. For α = 0, the microgel is fully swollen and no attraction is present, while, as α increases, the microgel progressively shrinks up to a collapsed state.

### Simulations of Single Objects and Calculated Observables

Molecular Dynamics simulations of single star polymers and single microgels are performed with the LAMMPS simulation package^26^ in the NVT ensemble using the stochastic velocity rescaling thermostat^27^ at a reduced constant temperature T* = *k*_B_ *T*/ϵ = 1, where *k*_B_ is the Boltzmann constant and *T* the temperature. We perform an initial equilibration run of 2 × 10^6^ *δ t*, with δ*t* = 0.002τ where $\tau =\sqrt{m\sigma^{2}/\epsilon }$ is the unit of time. Then, a production run is carried out for additional 10 × 10^6^ δ*t* steps, saving configurations every 5 × 10^4^ time steps. These are used to compute physical observables, such as the radius of gyration, defined as,

4 $$R_{g}=\left\langle {\left(\frac{1}{N_{m}}\sum_{i=1}^{N_{m}}{\left({\mathrm{r⃗}}_{i}-{\mathrm{r⃗}}_{\mathrm{cm}}\right)}^{2}\right)}^{1/2}\right\rangle$$

*N*_m_ being the number of beads per microgel, the density profile, defined as,

5 $$\rho \left(r\right)=\left\langle \overset{N_{m}}{\underset{i=1}{\sum }}\delta \left(\left|\mathrm{r⃗}-{\mathrm{r⃗}}_{i}\right|\right)\right\rangle$$

where *r* is the distance from the center of mass, and the form factor *P*(q⃗), defined as

6 $$P\left(\mathrm{q⃗}\right)=\left\langle \frac{1}{N_{m}}\overset{N_{m}}{\underset{i=1}{\sum }}\overset{N_{m}}{\underset{j=1}{\sum }}\exp\left[i\mathrm{q⃗}\cdot \left({\mathrm{r⃗}}_{i}-{\mathrm{r⃗}}_{j}\right)\right]\right\rangle$$

We also compute the hydrodynamic radius, following a protocol recently validated in ref 28 for PNIPAM-BIS microgels in comparison to available experiments. Here, we adapt the method to both star polymers and star-like microgels. In brief, *R*_H_ is calculated following Hubbard and Douglas^29^ as,

7 $$R_{H}=2{[\int_{0}^{\infty }\frac{1}{\sqrt{(a^{2}+\theta )\left(b^{2}+\theta \right)\left(c^{2}+\theta \right)}}d\theta ]}^{-1}$$

with *a*, *b,* and *c* the principal semiaxes of the effective ellipsoid representing the microgel at each equilibrium configuration. To evaluate this, we adopt two strategies: (i) we construct the convex hull for each equilibrium configuration and take the rigid ellipsoid that has the same gyration tensor of the convex hull;^21^ (ii) we evaluate the surface mesh using the α-shape method implemented in OVITO^30^ with probing radius = 12σ. Representative snapshots for the different macromolecular objects studied in this work are reported in the SI (Figure S1) to compare volume estimates obtained from convex hull and surface mesh methods. While the former is preferable for more compact objects since it does not depend on the choice of the probe radius, the latter is more suited to deal with macromolecules whose external surface is highly heterogeneous, such as in the presence of long dangling chains. The probed radius is thus chosen as the minimal one yielding no holes in the surface mesh of the particle. In the limit of very high probe radius, the two methods converge to each other. In the manuscript, we compare both estimates of *R*_H_ showing that they are mostly similar qualitatively, in order to provide robustness to our results. It is important to note that, for large macromolecules such as microgels or star polymers with large number of arms, it would be computationally very demanding to appropriately include hydrodynamic interactions, to reliably evaluate *R*_H_. This was done in ref 31 for stars with *f* up to 20. We thus use these previous results to benchmark the present calculations for low-*f* stars and then extending results for larger values of *f*.

From the instantaneous variation of the volume *V* of the effective ellipsoids, we are also able to compute the bulk modulus *K* = 1/κ_T_, inverse of the isothermal compressibility, as,

8 $$K=k_{B}T\frac{\left\langle V\right\rangle }{\left\langle V^{2}\right\rangle -\langle V\rangle^{2}}$$

enabling a direct comparison of the softness of the different examined particles.

### Calculation of Effective Potentials

To calculate the effective potentials, initial configurations of two star polymers or two microgel particles are prepared, randomly oriented with respect to each other, and placed at the center of the simulation box with side 600σ with periodic boundary conditions, at a relative distance **r**_**0**_ = (*x*_0_,0,0) from each other. We then wait for any transient to decay and perform a production run for at least 20 × 10^6^ δ*t* using a Langevin thermostat with friction coefficient ξ = 1. For all these calculations the solvophobic parameter was fixed to α = 0.0.

The effective potentials are calculated via the umbrella sampling technique,^32^ with a harmonic bias of energy *U*_*i*_ = 1/2 ϵ *k*_*i*_ [(*x* – *x*_0_)^2^ + *y* ^2^ + *z* ^2^] imposed over the natural variables of choice, namely the centers of mass for both microgels and stars or the central cores for stars. Hence, we sample the distances between the variables of choice every 250 δ*t* and run several simulations—windows—spanning *x*_0_ over the range of interest, at intervals of δ*x* = 1σ. A stiffness of *k*_*i*_ = 10 for the bias was utilized at short and intermediate distances, while *k*_*i*_ = 5 was used for the more distant cases. For each window, we calculate the biased probability distribution of finding the two particles at the imposed distance, then the harmonic contribution is removed, to calculate the unbiased probability *P*(*r*). Hence, the effective potential is finally calculated as

9 $$V_{\mathrm{eff}}\left(r\right)=-k_{B}T\;\ln P\left(r\right)+C$$

where *C* is set such that *V*(∞) = 0.

## Experimental Methods

The star-like microgels with 1% molar percentage of the cross-linker EGDMA are the same used in our previous work.^17^ They are synthesized using the same precipitation polymerization method as for standard microgels, just replacing BIS with EGDMA. All details on the synthesis are reported in ref 17.

In this work, we additionally report results of light-scattering measurements made by using a goniometer-based variable multiangle light scattering (V-MALS) instrument (LS Instruments, Switzerland), equipped with a He–Ne laser (120 mW power, 638 nm wavelength). The freeze-dried samples were dissolved in Milli-Q water at a concentration of 0.1 mg/mL. Measurements were performed as a function of temperature and scattering angle with the following protocol: a target temperature was set and, once stabilized, the system was allowed to thermally equilibrate for an additional 10 min. Subsequently, measurements were carried out across scattering angles ranging from 20° to 90° in increments of 2° (corresponding to a scattering wave vector range of 4.6 × 10^–3^ nm^–1^ ≤ *q* ≤ 1.9 × 10^–2^ nm^–1^). At each angle, four independent measurements of both the scattered intensity and the intensity autocorrelation function were collected. The hydrodynamic radius, *R*_H_, was determined from the intensity autocorrelation function using a second-order cumulant analysis. This yields the diffusion coefficient, *D*, which is converted to *R*_H_ via the Stokes–Einstein relation, *D* = *k*_B_ *T*/(6πη*R*_H_). The reported *R*_H_ for each temperature is the average value of measurements at scattering angles between 30° and 60°.

The radius of gyration, *R*_g_, was estimated by fitting the angular-dependent scattered intensity, *I*(*q*), after normalization by the scattered intensity of toluene, with the Guinier equation:

10 $$I\left(q\right)=I\left(0\right)\exp\left[-\frac{{\left(qR_{g}\right)}^{2}}{3}\right]$$

where *I*(0) is a constant that depends both on the scattering factor of a single particle and on the number of particles in the scattering volume. To estimate *R*_g_, we choose the range of *q* such that the relation 0.6 ≤ *qR*_g_ ≤ 1.5 is satisfied. Representative examples of the performed fits are reported in the SI (Figure S2).

## Results

### Effective Potential: Star Polymers

We start by reporting results for star polymers to make contact with literature and extend previous findings. Figure 1 shows the calculated effective potential for two different kinds of star polymers, namely a low-functionality star with *f* = 18 and *N*_*f*_ = 50 already reported in ref 33 and a relatively high-functionality star with *f* = 80 and *N*_*f*_ = 200, that was established to correspond to the best representation of *c* = 1% PNIPAM-EGDMA microgels investigated in ref 17. In doing this, we also explore a larger range of *f* and *N*_*f*_ with respect to available literature results.

**Figure 1:**
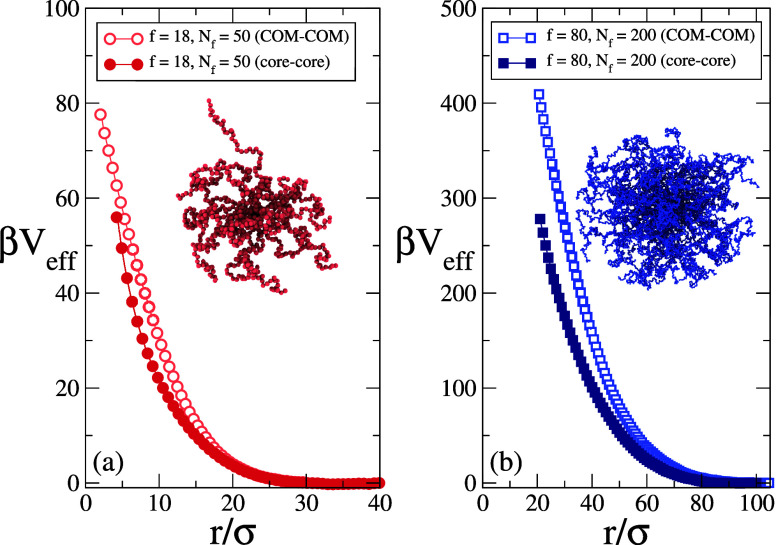
Effective potential β*V*_eff_ between star polymers with (a) *f* = 18, *N_f_* = 50 and (b) *f* = 80, *N_f_* = 200 versus both core–core distance *r*_core_ (full symbols) and COM-COM distances *r*_COM_ (open symbols). Lines are guides to the eye. The snapshots illustrate a typical single-molecule equilibrium configuration of the two systems. For the large star with *N_f_* = 200, the core is hardly visible.

Snapshots of both stars are reported in Figure 1 to visualize the simulated particles. Effective potentials are reported as a function of two possible distances: the most natural variable that expresses a distance between two stars, that is, the distance between the center points of the physical cores *r*_core_, and a more generic variable that applies to any macromolecule, such as microgels, namely the distance between the centers of mass *r*_COM_. As expected, the two potentials are almost identical at large distances, but significant deviations appear at short ones. Indeed, theory predicts a logarithmic divergence for the core–core interactions, which cannot physically overlap on approaching each other, while centers of mass are fictitious points which can also be empty of monomers. Although we cannot reach even shorter distances within the current simulations, we see that the slope of the potential between core distances gets steeper as *r*_core_ decreases, in agreement with expectations, since the two curves should eventually cross. In addition, deviations between core and COM representations are more evident when *f* is larger, due to the increased steepness of the repulsion, leading to stronger deviations between the two representations. To better grasp the microscopic differences between the two potentials, we report the behavior of *r*_COM_ versus *r*_core_ in our umbrella sampling simulations and their interplay in the SI (see Figures S3–S4 and related discussion).

We compare the calculated effective potentials with theoretical predictions in Figure 2, for core and COM distances in (a) and (b) panels, respectively. Starting with the core–core representation, we find excellent agreement between the numerical *V*_eff_ and the logarithmic ultrasoft potential, that is written in terms of an effective size σ_L_ following Likos and coworkers:^7^

11 V(r)=a0 f3/2{−ln(rσL)+11+f/2,r≤σL,σL/r1+f/2exp[−f(r−σL)2σL],r>σL

where *a*_0_ = 5/18 from the theory and we use it as a fit parameter to the numerical data. We find that the logarithmic behavior for *r* < σ_L_ satisfactorily describes the numerical data with a prefactor *a*_0_ in excellent agreement with the predicted value, for both stars as clearly visible in Figure 2a and in Table 1. These results validate the Witten and Pincus^6^ and Likos et al.^7^ approaches against numerical results for an unprecedented range of *f*, since in the literature so far only results up to *f* = 50 were provided.^8^

**Figure 2:**
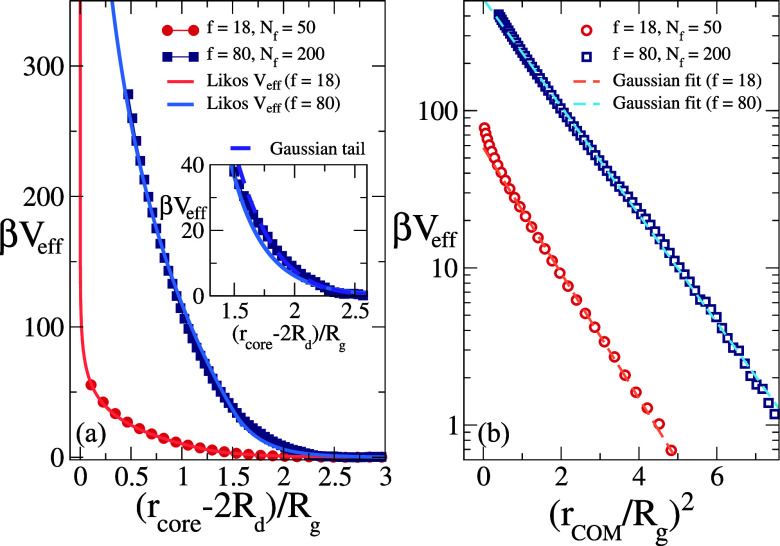
(a) Effective potential *V*_eff_ between star polymers with *f* = 18, *N_f_* = 50 and *f* = 80, *N_f_* = 200 with *r*_core_ as the natural variable. The diameter of the first corona of monomers near the core, 2*R*_d_, is subtracted in order to read the divergence in the origin. The two continuous lines represent the Likos potential fit with a Yukawa tail, as in eq 11. The theoretical prefactor 5/18 is treated as a parameter, reported as *a*_0_ in Table 1, in order to test the adherence to the *f* → ∞ limit. The inset additionally shows a fit with a Gaussian tail for *f* = 80. (b) Effective potential *V*_eff_ between the same star polymers with *r*_COM_ as the natural variable, normalized by *R*_g_. The dashed lines are Gaussian fits with parameters reported in Table 2.

**Table 1:** Likos Curve Fit Parameters, as in Eq 11, for the Effective Potential between Star Polymers as a Function of *r*_core_ ^a^

star	polymers	*a*_0_	σ_L_
*f* = 18	*N*_*f*_ = 50	0.2753	1.2
*f* = 80	*N*_*f*_ = 200	0.2796	1.5

a Note that the divergence has been shifted to *R* ∼ 0 using the variable *R* = *r*_core_ – 2*R*_d_, where 2*R*_d_ = 2*R*_c_ + *σ* is the Diameter of the first corona of monomers, and the fit parameters *σ*_L_ are given in units of (*r*_core_ – 2*R*_d_)/*R*_g_. For clarity, we also report *σ*_L_ in units of $\sigma :\sigma_{L}^{f=18}∼17.2\sigma ,\sigma_{L}^{f=80}∼54.8\sigma$ .

Regarding the large distance range, we recall that analytical predictions are not available. For this reason, Likos et al.^7^ postulated a crossover to a Yukawa tail, based on the favorable comparison with experimental form factors for *f* = 50. This tail is however incompatible with the Gaussian behavior observed for linear chains (*f* = 2), which led to the ansatz that for *f* < 10 the potential should cross over to a Gaussian form.^8^ This problem was later revisited by Hsu and Grassberger,^9^ who claimed a better description of the Gaussian form at all *f*.

Here, we show in Figure 2a that the Yukawa representation is qualitatively satisfactory for *f* = 18 but less accurate for *f* = 80 (see inset for a magnification at large distances). Indeed, it seems that a Gaussian tail, also shown in the inset of Figure 2a, reproduces the numerical data better.

Focusing instead on the COM representation, we report *V*_eff_(*r*_COM_) in Figure 2b for both stars, finding that a Gaussian description,^9^

12 $$V_{\mathrm{eff}}\left(r\right)=c_{f}e^{-d_{f}{\left(r/R_{g}\right)}^{2}}$$

successfully applies to almost all probed distances, except when the cores are very close to each other, with *c*_*f*_ setting the energy scale and *d*_*f*_ being related to the inverse of the standard deviation. The corresponding fit parameters are reported in Table 2, yet a direct comparison to the lattice results of Hsu and Grassberger is not easily possible. Notwithstanding this, the robustness of the Gaussian dependence on *r*_COM_ appears to be quite remarkable. It is however reassuring that at large distances the differences between the two representations of the potential are minor, thus not substantially affecting results, as also shown by the numerous validations of the Likos potential against experiments.^7,34–36^

**Table 2:** Gaussian Fit Parameters for the Effective Potential between Star Polymers as a Function of *r*_COM_

star	polymers	*d*_*f*_	*c*_*f*_
*f* = 18	*N*_*f*_ = 50	0.92	56.6
*f* = 80	*N*_*f*_ = 200	0.78	491

### Effective Potential: Standard and Star-Like Microgels

We now proceed by reporting the calculated effective potential between microgels, comparing star-like and standard ones at the same cross-linker concentration *c* = 1%. Results are reported in Figure 3 as a function of the natural variable *r*_COM_. We first observe in Figure 3a that *V*_eff_ is much more repulsive for standard microgels as compared to star-like ones, when they are compared at the same relative distance. This is due to the fact that the corona of the star-like microgel is much more extended and less compact, as visible from the snapshots also reported in the figure, which justifies the weaker interactions.

**Figure 3:**
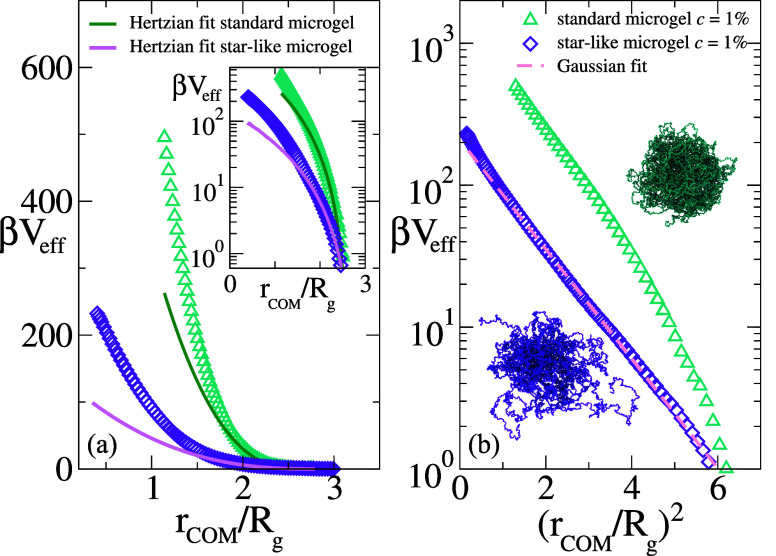
Left panel: effective potential *V*_eff_ between star-like microgels and standard microgels at a cross-linker concentration *c* = 1%. Hertzian fits are shown for both systems; the tail is highlighted in the log-scale inset. Right panel: the same potentials are shown on a Gaussian scale, where Gaussian profiles appear as straight lines. The Gaussian fit for the star-like microgel is shown as a dashed line. Fit parameters are reported in Table 3.

It is well-established that standard microgels under dilute conditions are a good model system for elastic spheres, interacting with the Hertzian potential, defined as

13 $$V\left(r\right)=\left\{ \begin{array}{ll} \epsilon_{H}{\left(1-\frac{r}{\sigma_{H}}\right)}^{5/2}, & \mathrm{if}\;r<\sigma_{H},\\ 0, & \mathrm{otherwise} \end{array}\right.$$

with an energy scale ϵ_H_, which depends on the elastic properties of the microgel, and an interaction range σ_H_, usually found to be coinciding with the hydrodynamic diameter.^37^ Here we confirm these results, as shown in Figure 3a, which also reports a Hertzian fit to the numerical results, whose parameters are provided in Table 3. In agreement with previous results,^21^ the Hertzian description holds up to a moderate range of distances, below which the calculated potential is more repulsive, due to the heterogeneous core–corona structure of the microgel, which increases the internal elastic response of the particles at strong compression.^38^ A similar description also applies to the star-like microgel, albeit the deviations from the Hertzian form appear to be more pronounced. We argue that the Hertzian behavior for the star-like microgels effective potential is only apparent and that instead a Gaussian description is valid for most probed distances. This is shown in Figure 3b, where a Gaussian fit is seen to apply to the numerical data for several orders of magnitude. The same does not clearly hold for the regular microgel, which shows clear deviations from a Gaussian shape. Interestingly, the resulting Gaussian fit parameters, especially *d*_*f*_, also reported in Table 3, are rather similar to those found for star polymers, reported in Table 2.

**Table 3:** Fit Parameters for Star-Like and Standard Microgels Effective Potential^a^

microgel	type	ϵ_H_	σ_H_	*c*_*f*_	*d*_*f*_
*star-like*	*c* = 1%	138.5	103.3	209	0.89
*standard*	*c* = 1%	1092	73.6	-	-

a Both Hertzian and Gaussian parameters are reported. Note that *σ*_H_ is given in units of the monomer size *σ*. As a reference, *R*_g_ is found to be 37.0*σ* and 28.1*σ* for star-like and standard microgels, respectively.

### Comparison between Star Polymers and Star-Like Microgels

After having discussed star polymers and microgels separately, we report a direct comparison between the effective potential for star-like microgels and for star polymers with *f* = 80, *N*_*f*_ = 200 as a function of *r*_COM_. Given their similar structure, already documented in ref 17, we would also expect their interaction potentials to be similar. However, although sharing a similar Gaussian profile given by the comparable values of *d*_*f*_ (see Table 2), we find that *V*_eff_ between star polymers is much more repulsive than the corresponding one for star-like microgels, as shown in Figure 4a. This originates from the much stronger local crowding of chain monomers near the cores of the stars, as compared to the same taking place around the microgel cross-linkers. This effect is clearly visible in the snapshots reported in Figure 4b, taken at a comparable distance. Clearly, the microgels are far less dense in monomers in their inner part, leading them to interact with a softer repulsion. Given this observation, which stems from the full coverage of the cores of the stars, as opposed to the looser structure around the cross-linkers of the microgel, we reduce the number of arms of the star polymer, while keeping *R*_c_ fixed. We thus build a star polymer with a partially covered surface of the core, choosing *f* = 46 which corresponds to an effective coverage fraction γ ∼ 0.57. For this specific value, we remarkably find an effective potential that is virtually indistinguishable from that between star-like microgels, as also shown in Figure 4a and in the relative inset. Indeed, the snapshots between the partially covered stars are very similar to those between star-like microgels. Hence, the local monomer density around the cores or the cross-linkers appears to be the main parameter distinguishing pure stars from star-like microgels.

**Figure 4:**
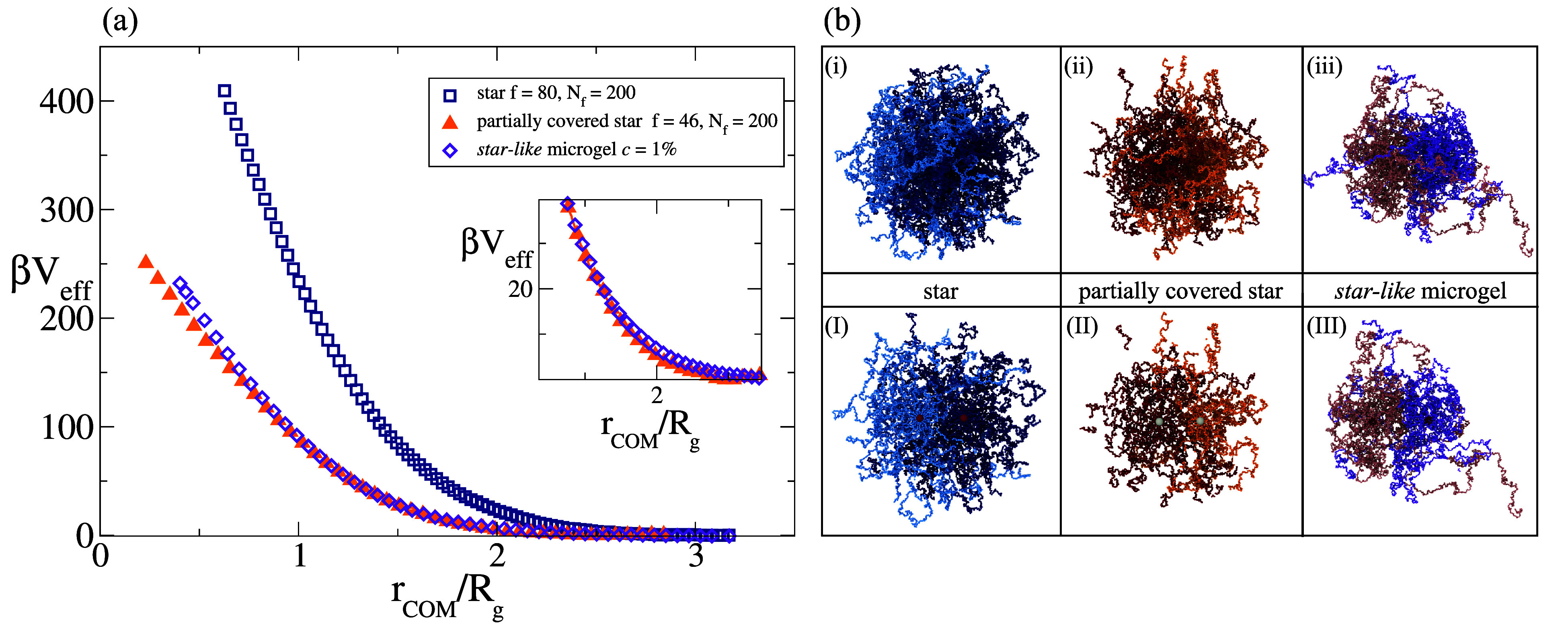
(a) Effective potential β*V*_eff_ between *c* = 1% star-like microgel, between star polymers with *f* = 80, *N_f_* = 200, *R*_c_ = 2.2 σ and between partially covered stars with *f* = 46,*N_f_* = 200, *R*_c_ = 2.2σ, as a function of *r*_COM_ rescaled by the respective gyration radii. The inset enlarges the short distance behavior; (b) snapshots between two of each macromolecules at a distance *r*_COM_/*R*_g_ ∼ 0.6 to visually explain the differences observed in the effective potentials. The top row shows the full macromolecules, while the bottom one reports the corresponding slices. Note that the cores of the stars as well as the cross-linkers of the star-like microgels have a different color.

### About Star Polymers with Reduced Core Surface Coverage

The effect of the core surface coverage on the physical properties of a star has not been investigated in detail before. We thus report here the variation of the structure, quantified by the density profile of the particles, calculated with respect to the centers of mass, in Figure 5. We see that all profiles, namely that for a pure star with *f* = 80 and full coverage (*R*_c_ = 2.2σ), for a partially covered star with *f* = 46 (*R*_c_ = 2.2σ), and for the star-like microgels, are all very similar. As expected, also the form factors are similar, although the star with full coverage clearly has a more pronounced peak, as visible in the inset. This is indicative of a more crowded zone near the core, given its high functionality, that leads to the discussed more repulsive interactions. On the other hand, the partially covered star and the star-like microgel share a similar peak profile.

**Figure 5:**
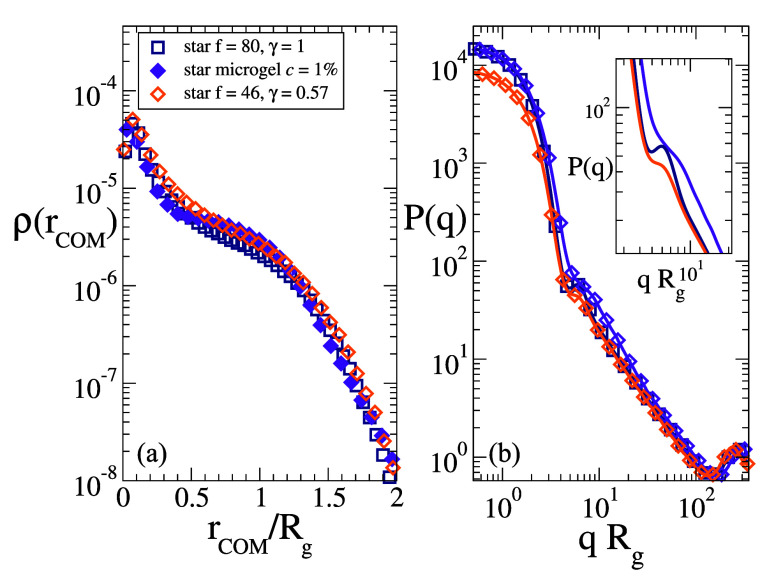
(a) Density profiles ρ­(*r*_COM_) and (b) form factors *P*(*q*) for the star polymer with *f* = 80, *N_f_* = 200, for the *star-like* microgel with *c* = 1%, and for the partially covered star with *f* = 46, *N_f_* = 200. Data in panel (a) are normalized to yield a volume integral equal to 1, while in (b) they are only scaled by the respective gyration radii. The inset shows a zoom-in of the peak profiles.

So far, we have adjusted the coverage fraction γ by keeping the core size fixed while varying the star polymer functionality *f*, thus probing the dependence γ = γ­(*f*). It is also interesting to investigate whether, at fixed functionality *f*, the core size has a significant effect on the star polymer interactions. To this end, we consider three systems in which *f* and *N*_*f*_ are fixed, while γ = γ­(*R*_c_) is tuned by changing the core radius *R*_c_. In particular, we use *R*_c_ = 1.7 σ (γ ∼ 1), *R*_c_ = 2.2 σ (γ ∼ 0.57), and *R*_c_ = 4 σ (γ ∼ 0.18). The latter value is very similar to that obtained for star-like microgels. Indeed, the cross-linker-rich core radius can be estimated from the ensemble-averaged *R*_g_ of the cross-linkers only and converted into the radius of an equivalent solid sphere, obtaining *R*_c_ ∼ 3.5σ. For these systems, we calculate the two-body mean force as a function of the core–core distance, as commonly done for star polymers.^39^ The results are plotted in Figure 6, showing that over a wide range of distances, the mean-force curves as a function of *r*_core_ are nearly indistinguishable among each other despite the change of coverage (see panel (a)).

**Figure 6:**
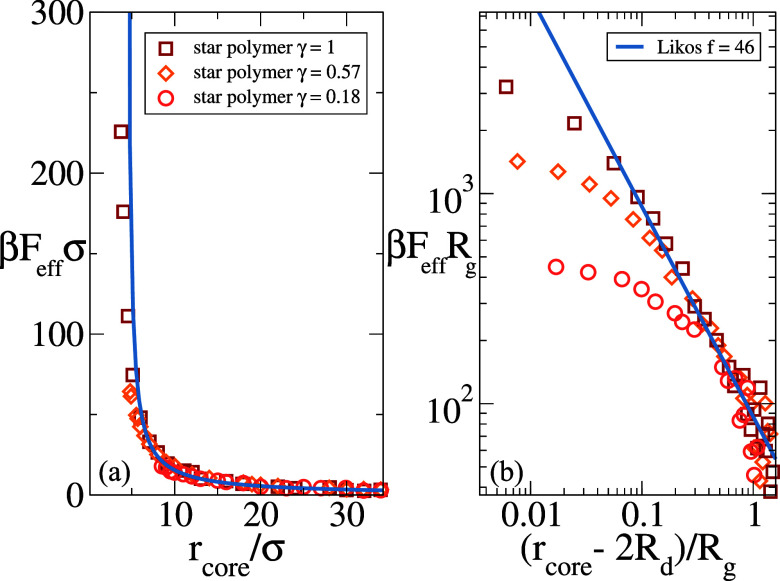
Effective mean force β*F*_eff_ for three star polymers with fixed *f* = 46, *N_f_* = 200 and different coverage fractions γ, achieved by changing *R*_c_ as explained in the text. In panel (a) data are shown as a function of *r*_core_, while in panel (b) this is shifted by 2*R*_d_ similar to previous studies.^7^ The line in both plots represents the negative gradient of the Likos potential in eq 11, i.e., the force. In panel (a), this is set to diverge for the fully covered case.

This weak sensitivity to *R*_c_ is expected in the regime *N*_*f*_ ≫ *f* ^1/2^, where the arms are long enough that the dominant contribution comes from the swollen corona and the interaction is set mainly by the overall number of arms (and the outer length scale), rather than by microscopic details of the grafting pattern. Small but systematic differences only emerge at short separations, where finite-core effects become relevant and the mapping onto the asymptotic star potential deteriorates. To visualize this, we plot in panel (b) the same data as a function of (*r*_core_ – 2*R*_d_)/*R*_g_ in a log–log representation. As expected, deviations from the logarithmic divergence increase with increasing core radius. This trend is consistent with a reduced number of chains effectively participating in the contact region when γ < 1, which lowers local crowding, and thus the entropic penalty, at a given core separation.

These results show that the effect of the coverage fraction is only relevant close to the core, thus explaining why a star-like microgel has similar interactions to those of a pure star, although less repulsive (see Figure 4). Indeed, the coverage fraction of the star-like microgel is lower than that of the stars, but not so much for low *c*, where the cross-linkers-rich core is small. Instead, for PNIPAM-EGDMA microgels at higher *c*, where the core size becomes significant as shown in ref 17, the star-like behavior is already lost in the overall structure, so it will be interesting to see what happens in terms of interactions.

### Properties across the Volume Phase Transition

To provide a comprehensive characterization of the different macromolecules and to explore their behavior upon increasing temperature, we also carry out simulations in the presence of the solvophobic potential (eq 3) varying the parameter α, which plays the role of an effective temperature. The VPT transition for PNIPAM-based microgels occurs for α ∼ 0.65. However, ref 17 has shown that the VPT occurs much more sharply for star-like microgels than for regular ones, as also captured by our numerical model. This is due to the strong decoupling between the arms and the core that occurs in star-like objects, as opposed to cross-linked microgels, whose greater connectivity smoothens the transition.

We characterize the different systems by looking at two observables as a function of temperature, namely the ratio between gyration and hydrodynamic radius *R*_g_/*R*_H_, also in comparison to new experimental measurements, as well as the bulk modulus which is a measure of the softness of the particles.

#### Ratio between Gyration and Hydrodynamic Radius

The ratio between gyration and hydrodynamic radius is often used to characterize the mass distribution within a macromolecule.^28^ In particular, while *R*_g_ quantifies the polymeric distribution around the center of mass, the hydrodynamic radius is more sensitive to the presence of the outer chains, contributing to the diffusion of the particle. Hence, while for homogeneous spheres, *R*_g_/*R*_H_ is found to be $\sqrt{3/5}$ , for star polymers with low arm number values greater than 1 have been reported.^40–43^ Regular microgels have been reported to have a value of this ratio close to 0.5–0.6, tending to the HS value as temperature increases and the particle collapses.^28,44^ It is then important to estimate this ratio also for star-like microgels, in order to further assess their similarity to star polymers.

To do so, it is important to first establish a meaningful way to estimate the hydrodynamic radius in simulations without explicit hydrodynamics, to avoid the high computational cost associated with explicitly accounting for them in such complex macromolecules. We thus resort to a method recently reported for regular microgels, as described in Methods. To validate this procedure in the case of star polymers and to extend current calculations to large values of *f*, needed to compare to star-like microgels, we first report *R*_g_/*R*_H_ in good solvent for star polymers with different functionalities in Figure 7. The numerical calculations are performed using two different approximations to estimate the volume of the particle, the convex hull and the surface mesh as explained in Methods and also illustrated in the SI (see Figure S1). Both approaches yield qualitatively similar results, where the ratio decreases with functionality, also in agreement with available numerical estimates for low *f* including explicit hydrodynamic interactions (HI). In ref 31, two approaches based on Multi-Particle Collision Dynamics (MPCD)^45^ were compared: a monomer-resolved study vs a penetrable soft colloid model, both yielding qualitatively similar results. It is clear that, while for small *f* the ratio *R*_g_/*R*_H_ strongly exceeds 1 in the presence of HI, it decreases upon increasing number of arms. This is expected, since, for *f* → ∞, one should recover the HS limit. We find that the present calculations based on the surface mesh are quantitatively closer to the monomer-resolved results in the presence of HI, and although our estimate is only qualitative, the trend with *f* is robust and scales similarly to the HI results.^31^

**Figure 7:**
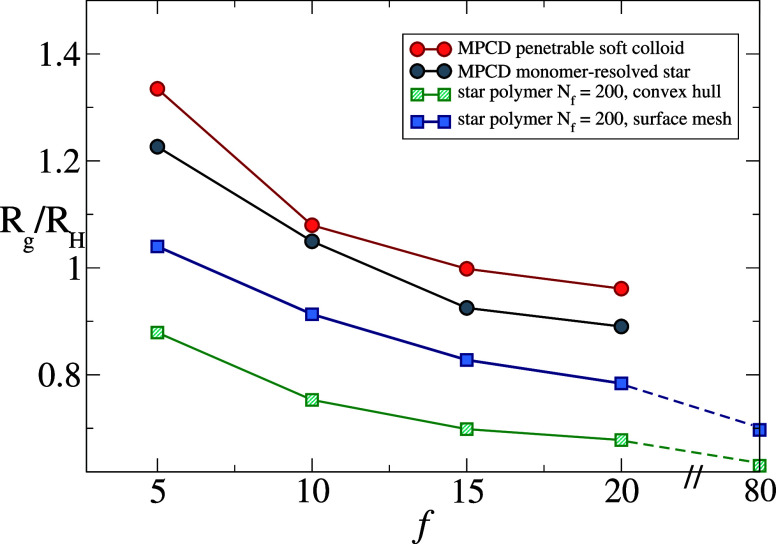
Ratio between gyration and hydrodynamic radius in good solvent (α = 0) for star polymers as a function of *f* with the two methodologies (convex hull vs surface mesh) to calculate *R*_H_. Comparison with MPCD results from ref 31.

Having established the reliability of our calculations, we next report the behavior of *R*_g_/*R*_H_ as a function of the solvophobic parameter α. First of all, we focus on star polymers and find that a very different trend with α takes place for low versus high number of arms, as shown in Figure 8. Indeed, while *R*_g_/*R*_H_ decreases monotonically with α for low-*f* stars, for high-*f* star polymers the ratio is found to first decrease and then increase again, exhibiting a characteristic minimum associated with the VPT transition, already observed in regular microgels.^28,44^ Interestingly, in those cases, the minimum was attributed to the presence of charges on the external microgel corona, which delays the collapse of the outer chains with respect to the core and was not observed in simulations of neutral microgels, albeit with a larger cross-linker concentration and only for the convex hull calculation.^28^ Here, we find that the minimum exists for neutral stars, independently of the method used. This is probably due to the higher degree of heterogeneity of the particles, whose outer chains may undergo deswelling earlier than the inner ones.

**Figure 8:**
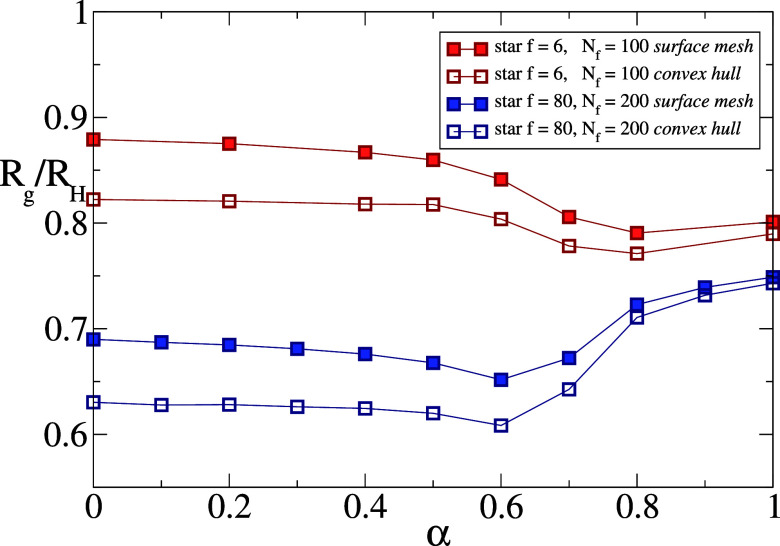
*R*_g_/*R*_H_ for two star polymers, one with low functionality *f* = 6, *N_f_* = 100, one with high functionality *f* = 80, *N_f_* = 200. The graph shows how the ratio *R*_g_/*R*_H_ decreases going from good solvent to bad solvent for the low functionality star, increases for *f* = 80, exhibiting a minimum at the VPT transition. The surface mesh and convex hull curves yield the same qualitative behavior.

Next, we compare the behavior of *R*_g_/*R*_H_ for the star polymer with *f* = 80 to the corresponding one for the star-like microgel with *c* = 1%. In order to have a full picture, we also show the ratio calculated for a regular microgel, again with *c* = 1%, and for the partially covered star with *f* = 46, as shown in Figure 9. We find that, while all data are generally similar, star-like microgel is the one showing the sharpest growth as α is further increased. This is especially true when compared to the regular microgels, reflecting the sharpness of the VPT of PNIPAM-EGDMA star-like microgels already observed by DLS.^17^ In addition, the fully covered and the partially covered stars seem to bracket the values of the ratio *R*_g_/*R*_H_ for the star-like microgel. All three star-like systems display a very pronounced minimum in *R*_g_/*R*_H_, probably due to the cooperative nature of the arms undergoing the VPT as compared to the fixed core. In contrast, for the standard microgel the minimum is barely detected. Note that, with respect to the data reported in ref 28, where the minimum was not found for neutral microgels, here we find it for a slightly lower *c* and using the surface mesh method. Indeed, the minimum tends to disappear when using the convex hull for standard microgels, as shown in the SI (Figure S7).

**Figure 9:**
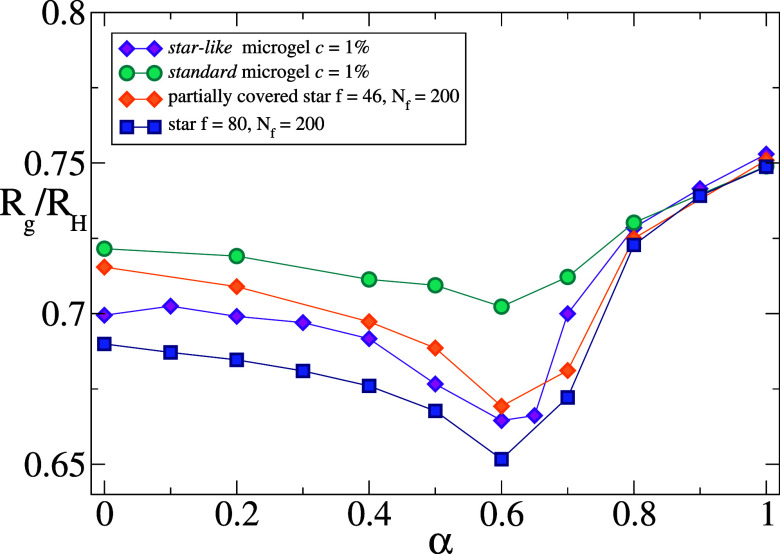
Ratio *R*_g_/*R*_H_ for a *star-like* microgel with *c* = 1%, for a *standard* microgel also with *c* = 1%, for the fully covered star (*f* = 80, *N_f_* = 200) and for a partially covered star with *f* = 46 and *N_f_* = 200 (γ ∼ 0.57). The latter is the one that best reproduces the effective potential of the *star-like* microgel (see Figure 4). The ratio is estimated in all cases using the surface mesh method for the hydrodynamic radius.

To verify these predictions, in Figure 10 we directly compare *R*_g_/*R*_H_ for the star-like microgel with experimental results, obtained by SLS and DLS experiments on PNIPAM-EGDMA microgels with *c* = 1%. The experimental swelling curves for *R*_g_ (SLS) and *R*_H_ (DLS) are also shown in the SI (Figure S5), together with the corresponding ones obtained by simulations (Figure S6). We find that the direct comparison of experiments and simulations is in qualitative agreement. As expected, the minimum of *R*_g_/*R*_H_ is much more pronounced in experiments, due to the presence of electrostatic effects introduced by the ionic initiator,^28^ not considered for simplicity in the simulations. It is confirmed also in experiments that for large enough number of arms the ratio does not exceed 1, at swollen conditions, and tends to the HS value at large temperatures. Again, the sharpness of the VPT is evident from the experimental data.

**Figure 10:**
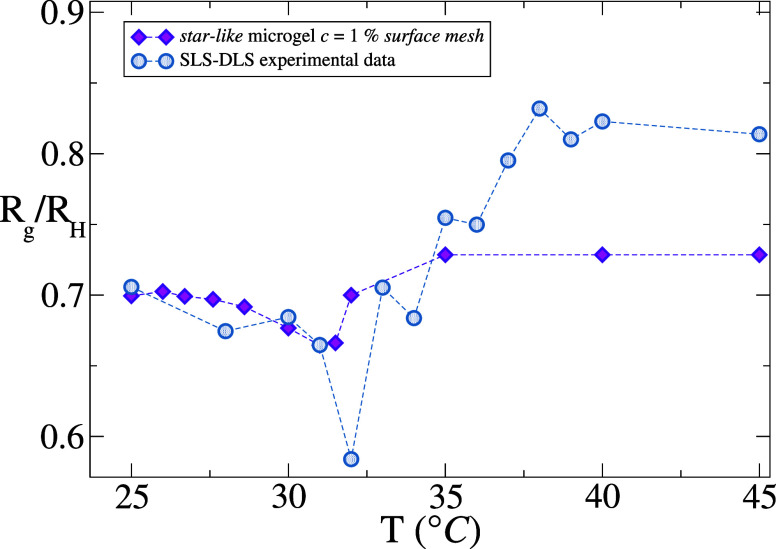
Comparison for *R*_g_/*R*_H_ between *in silico star-like* microgel and SLS-DLS experimental data. Dashed lines are to guide the eye.

#### Bulk Modulus

Finally, to provide an estimate of the softness of the particles we also calculate their bulk modulus *K* as a function of effective temperature. This is simply estimated from the volume fluctuations, as done in previous works.^21^ This is reported in Figure 11, which shows that the star-like microgel, as well as the partially covered star, displays a bulk modulus almost 1 order of magnitude lower than regular microgels with the same degree of cross-linking. No minimum in *K* is detected at the VPT, for any system. Finally at large α, under collapsed conditions, all moduli tend to coincide. Similar results are obtained when using the convex hull method (see Figure S8), showing qualitatively similar results. Given the large softness of star-like microgels, it is instructive to compare the calculated value of *K* for α = 0 with the corresponding one estimated for ultralow-cross-linked (ULC) microgels of comparable numerical size.^46^ ULC microgels are synthesized in the absence of cross-linkers and are considered to be the softest existing type of microgels. Their bulk modulus ∼10^–3^ is thus confirmed to be slightly lower than for star-like microgels, but only by a factor ∼2, as compared to the order of magnitude difference found with respect to regular microgels.

**Figure 11:**
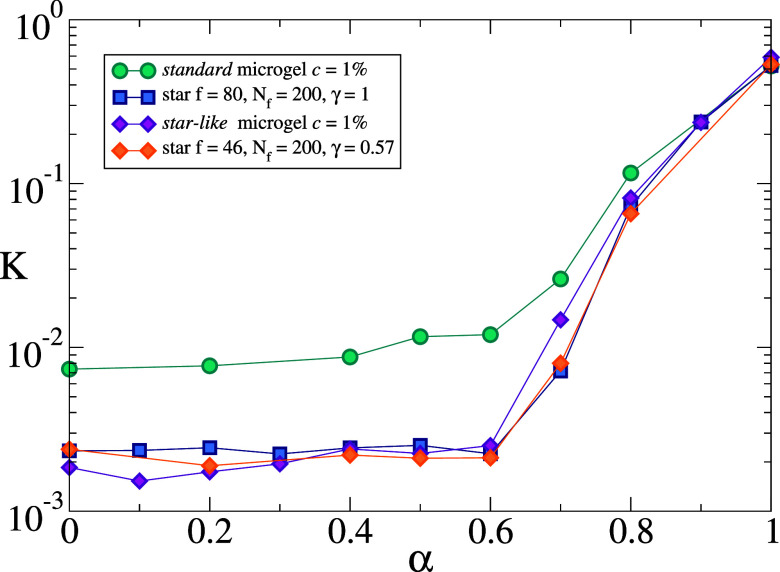
Bulk modulus *K* in units of *k*_B_ *T*/σ^3^ as a function of effective temperature α, obtained using the surface mesh method to estimate the volume.

## Conclusions

In this work we reported extensive numerical simulations of star-like microgels with *c* = 1%, easily realizable in the laboratory simply by substituting BIS with EGDMA cross-linkers in the chemical synthesis,^17^ and compared their structural properties and their effective potentials to those of star polymers and standard microgels. Through a detailed analysis of all these quantities, we have demonstrated that star-like microgels with low degree of cross-linking are a different class of soft colloids with respect to standard microgels. In particular, they are much softer, as demonstrated by their elastic moduli roughly 1 order of magnitude lower, and they display a Gaussian effective potential in a wide range of relative distances, as opposed to the Hertzian-like one of regular microgels. The latter feature appears to be also very different from recent results for ULC microgels, which seem not to obey any effective pair potential due to their extreme deformability.^46^ Thus, despite a similar value of the bulk modulus, star-like microgels appear to be also distinguished from ULC microgels.

On the other hand, the characteristics evidenced in this work actually establish a deep connection between star-like microgels and star polymers, since they share the same functional form of the effective potential, as well as a similar behavior of the elastic moduli and of the ratio between gyration and hydrodynamic radii. However, we also found that ideal stars with full core coverage experience a much more repulsive interaction than star-like microgels, due to the larger density of monomers in between the two cores. To improve the similarity between star-like microgels and star polymers, we thus considered stars with a partial core coverage, finding remarkable agreement between the two also in terms of quantitative effective potential.

These findings align with expectations that microgels synthesized by a precipitation polymerization process around a tiny central core do not have the ability to fill the core coverage, thus remaining partially naked. Given that the partial core coverage was not previously considered in the literature, we also provided evidence that it is a minor difference in the topology of the particles, not affecting the structural properties of the stars and the vast results already established in the literature, including the logarithmic dependence of the interparticle potential upon core–core distance. It will be interesting in the future to compare the present results with alternative types of microgels with star-like characteristics, resulting from a more complex synthetic method developed in the group of Kanaoka,^47^ that have not yet been described in terms of theoretical models.

To conclude, star-like microgels are viable alternatives to star polymers, with very similar single-particle properties and two-body effective interactions, that will be extremely interesting to investigate in the future. Indeed, the coupling of their ultrasoft character with their enhanced softness, as compared to standard microgels, may provide interesting results for phase behavior and rheological response in dense suspensions.

## Supplementary Material



## Notes

Representative snapshots of simulated macromolecules; experimental measure of *R*_g_; difference in the choice of variable (COM vs core) for effective interactions of star polymers; numerical and experimental swelling curves; numerical results from the convex hull approach (PDF)
