# Supplementary material for: Star-Like Microgels vs Star Polymers: Similarities and Differences
Source: Macromolecules. 2026 May 5;59(10):5980–90. doi: 10.1021/acs.macromol.6c00287 (PMC13217620; doi:10.1021/acs.macromol.6c00287)
Supplement: Supplementary file 1 [file ma6c00287_si_001.pdf]

Supplementary Information for: Star-like microgels vs star polymers: similarities and differences

Tommaso Papetti,*,†,‡ Elisa Ballin,†,‡ Francesco Brasili,‡,† and Emanuela Zaccarelli*,‡,†

†*Department of Physics, Sapienza University of Rome, Piazzale Aldo Moro 2, 00185, Roma, Italy*

‡*CNR Institute of Complex Systems, Uos Sapienza, Piazzale Aldo Moro 2, 00185, Roma, Italy*

E-mail: tommaso.papetti@uniroma1.it; emanuela.zaccarelli@cnr.it

Representative Snapshots

In Fig. S1 we present representative snapshots for the different types of soft particles analyzed in the main text, to illustrate how we estimate their volume. In particular, each snapshot compares the volumes obtained with the convex hull approximation (top row) and the surface mesh approach (bottom row), along with the corresponding effective ellipsoids.

The Figure shows that the ellipsoids obtained from the surface mesh method are systematically smaller than those derived from the convex hull approach. This difference is particularly evident for the star-like microgel (c), highlighting that the convex hull method is especially sensitive to conformations featuring occasional long outer chains. In addition,

the star-like microgel appears to be most asymmetric among the studied particles, again due to the presence of the long outer chains. Given that the surface mesh seems to give a more accurate estimate of the particle volume, we then mostly use this method in the main text.

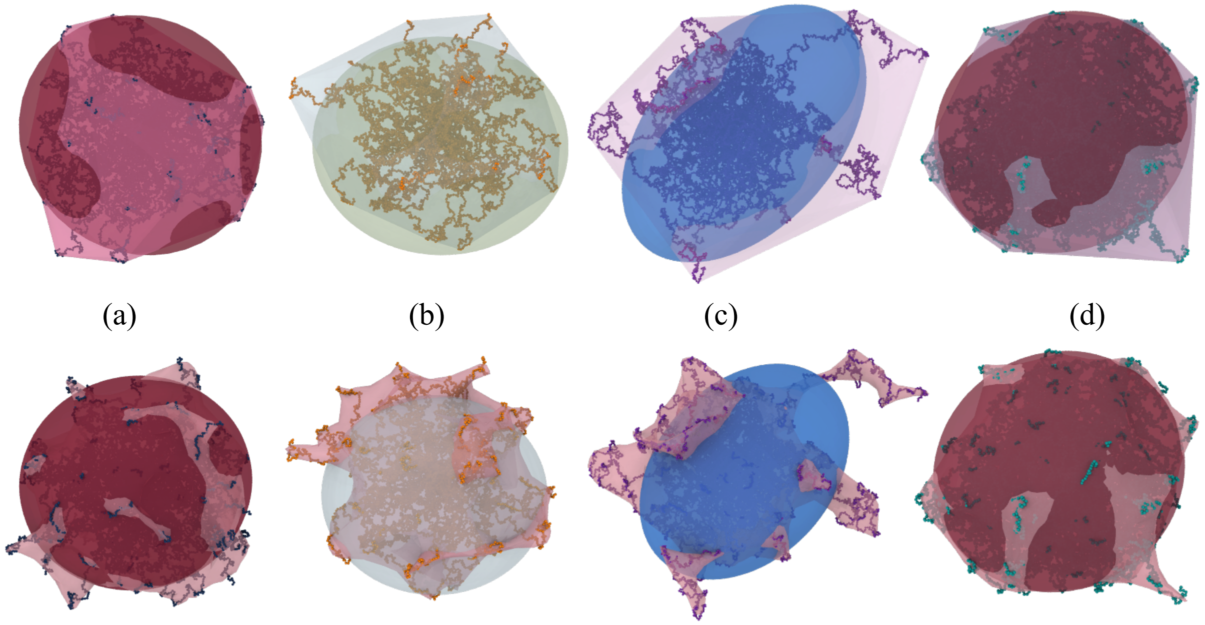

Figure S1: Snapshots for various particles analyzed in the paper. The top row represents the particles together with their convex hull mesh and relative effective ellipsoid, the bottom row is analogous but referring to the surface mesh method. (a): star polymer with $f = 80$, $N_f = 200$; (b): partially covered star polymer with $f = 46$, $N_f = 200$, $\gamma = 0.57$; (c): star-like microgel with $c = 1\%$ and (d) standard microgel with $c = 1\%$.

Experimental measure of R_g

As explained in the main text, the radius of gyration R_g was measured from static light-scattering (SLS) by fitting the angular-dependent scattered intensity with the Guinier form:

$$I(q) = I(0) \exp\left[-\frac{(qR_\mathrm{g})^2}{3}\right]. \tag{S1}$$

The measured intensity $I(q)$ was normalized by the scattered intensity of Toluene, $I_{\text{toluene}}(q)$, and analyzed in the Guinier representation, i.e., by plotting $\ln[I(q)/I_{\text{toluene}}(q)]$ as a function of q^2. Representative fits of the SLS data at different temperatures are reported in Fig. S2.

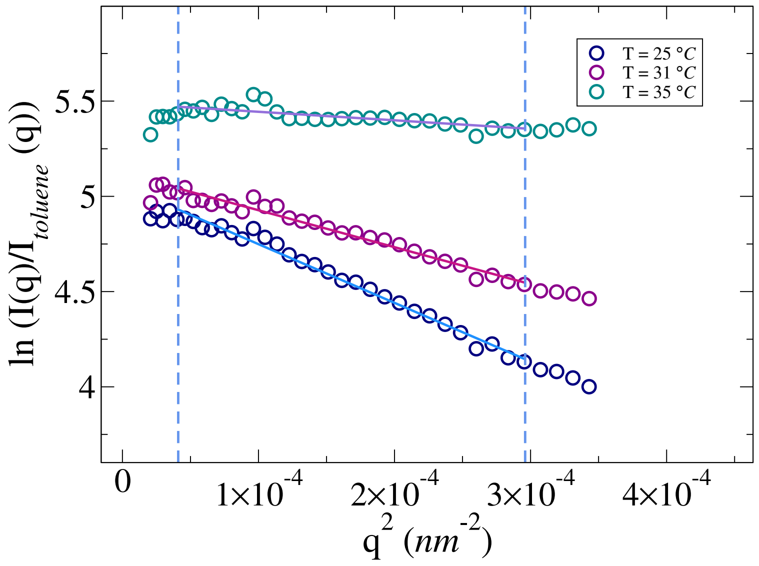

Figure S2: Selected Guinier fits for the estimation of R_{g} from SLS data at three studied temperatures for PNIPAM-EGDMA microgels with c=1%. Vertical dashed lines are for visual reference to the fit interval.

Difference in the choice of variable (COM vs core) for effective interactions of star polymers

The choice of variable has a significant impact on the effective two-body interaction between star polymers. Namely, the potentials expressed as a function of the distance between the cores or between the centers of mass have different functional forms. This is demonstrated by our umbrella sampling simulations, which can be perfomed biasing one or the other variable. As shown in Fig. S3, where we plot, for each umbrella window, the mean value of the unbiased distance versus the biased one, the two constraints used in the context of umbrella sampling technique select qualitatively different regions of configurational space over a substantial range of separations.

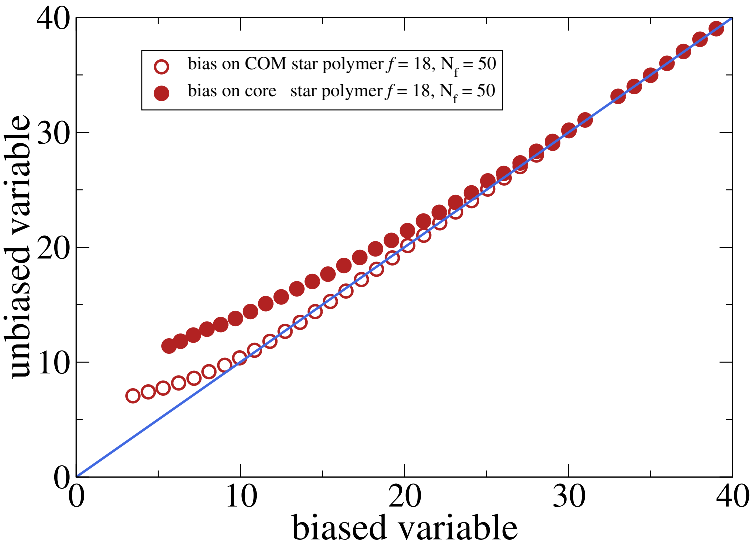

Figure S3: Behavior of the mean separation between natural variables for the umbrella sampling windows of the star polymer with $f = 18$, $N_f = 50$. The x-axes represents the biased variable, i.e., the natural variable chosen for the potential - either r_{COM} or r_{core} - while the y-axes shows the mean distance of the other respective variable averaged over the simulation for each of the windows. The blue line is the bisector.

This asymmetry is twofold: the separation from the bisector is smaller for the COMs bias and takes place at closer separations, while the bias-on-cores line separates widely from the bisector and early on, beginning even at $r \sim 2.4R_{\mathrm{g}}$, indicating that the conditional distributions $P(r_{\mathrm{COM}} \mid r_{\mathrm{core}})$ and $P(r_{\mathrm{core}} \mid r_{\mathrm{COM}})$ are broad and, crucially, not equivalent in the range where the two macromolecules start to mutually deform and interpenetrate. As a consequence, a simple functional mapping between the two distances such as $V(r_{\mathrm{COM}}) = V[r_{\mathrm{core}}(r_{\mathrm{COM}})]$ cannot be used to reconcile the difference.

The microscopic origin of these deviations is illustrated by the snapshots in Fig. S4. Indeed, constraining r_{core} directly controls the separation of the anchoring regions of the chains, the cores, where crowding is strongest, while leaving the mass distribution of the arms free to reorganize around that constraint, so that they move away from the central zone. In contrast, constraining r_{COM} can be satisfied through internal rearrangements of the chains: the stars redistribute their mass, so that the centers of mass approach each other without requiring an equally small core–core distance. This results in a more crowded

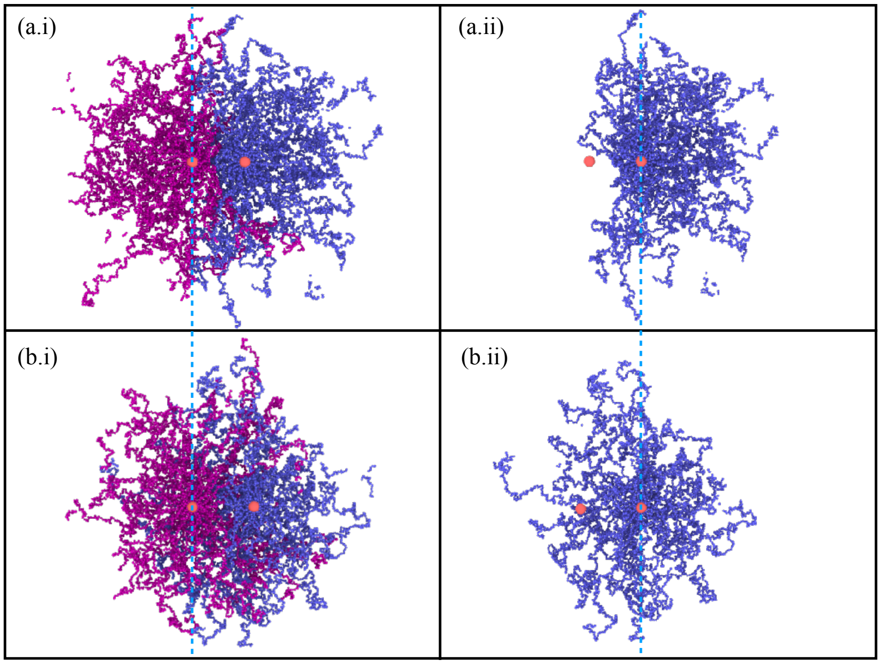

Figure S4: Equilibrium snapshots of two star polymers with $f = 80$, $N_f = 200$ taken during the umbrella sampling window at a separation value 20 in each of the two variables: (a) r_{core} and (b) r_{COM}. The second column (ii) shows the detail of only a single particle in the same configuration, hiding the other particle except its core. Note that the cores in the second row are found at a separation larger than 20σ, since the bias is on r_{COM}. The vertical dashed lines are guides to the eye.

monomer-rich central region when the bias is applied to the COM, and consequently V_{eff} (see Fig.1 of the main text) is more repulsive over a substantial range of separations. Clearly, the two potentials eventually cross: the cores are physical points and the potential diverges upon core contact, whereas the centers of mass are fictitious points, and their overlap is, in principle, allowed.

Swelling curves

For completeness we also report the swelling curves for R_{H} from DLS data and R_{g} from SLS data in Fig. S5 and compare them with the numerical swelling curves of Fig.S6. The latter also reports data for the additional types of particles studied in the simulations.

Notably, for star-like microgels, in both experiments and simulations, the reduction of R_{H}

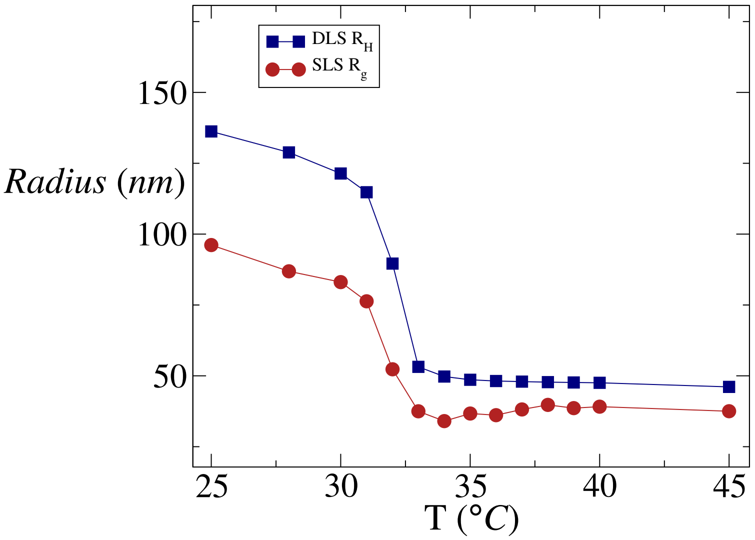

Figure S5: Swelling curves for the SLS (R_g) and DLS (R_H) data of the star microgel with $c = 1\%$ concentration of crosslinkers.

is more pronounced than that of R_g, indicating that the hydrodynamic size is particularly sensitive to the rearrangement of the outer, more dilute corona.

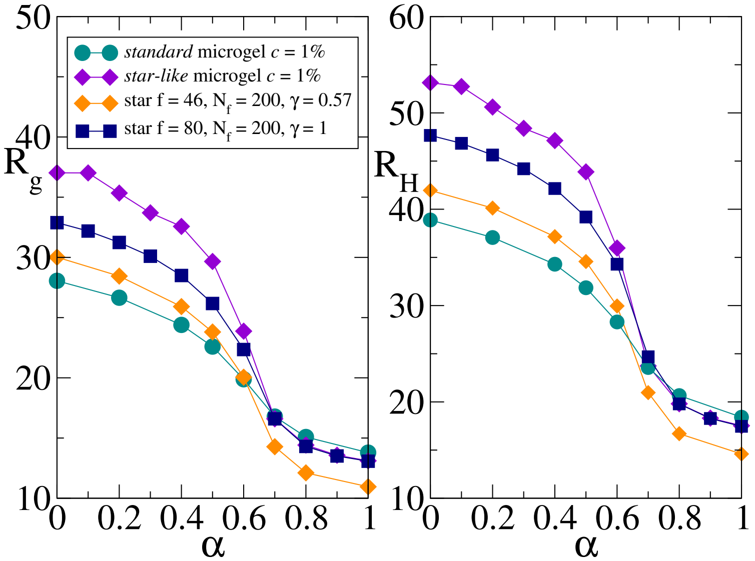

Figure S6: Swelling curves for the different simulated particles. Here, R_H is calculated within the surface mesh method.

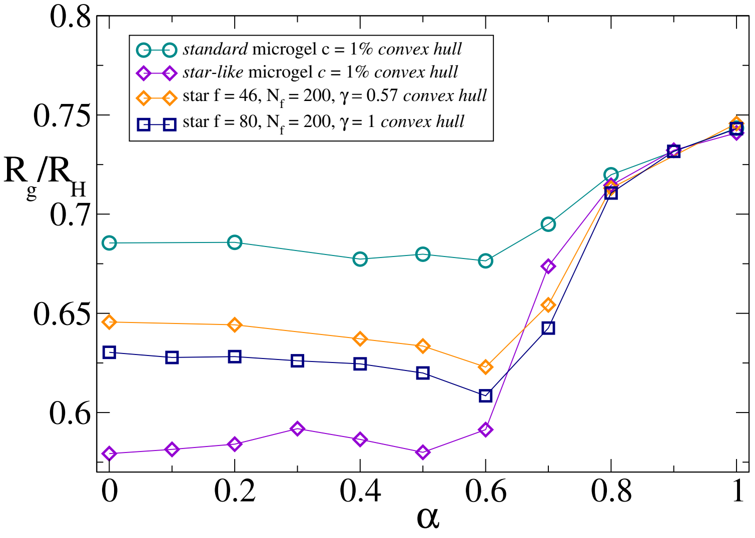

Figure S7: Ratio $R_{\mathrm{g}}/R_{\mathrm{H}}$ for the *star-like* microgel with $c = 1\%$, for the standard microgel also with $c = 1\%$, for the fully covered star ($f = 80$, $N_f = 200$) and for the partially covered star with $f = 46$ and $N_f = 200$ ($\gamma \sim 0.57$). R_{H} is calculated with the convex hull method.

Numerical results from the convex hull approach

Finally, we report the convex-hull-based estimates of the ratio $R_{\mathrm{g}}/R_{\mathrm{H}}$ and of the bulk modulus, complementing the surface-mesh results discussed in the main text.

The ratio $R_{\mathrm{g}}/R_{\mathrm{H}}$ obtained by computing R_{H} from the convex hull is shown in Fig. S7. While the overall evolution with α is consistent with a progressive collapse across the VPT region, the convex hull amplifies differences between architectures in the good-solvent regime: the star-like microgel displays the strongest deviation because even a small number of extended chains can substantially enlarge the convex envelope and thus increase the inferred R_{H}.

Figure S8 reports the bulk modulus K as a function of α when the particle volume is estimated from the convex hull. The transition region is still clearly identified by the sharp increase of K upon collapse. However, the star-like microgel yields systematically smaller K values with respect to the other systems. This is consistent with the convex hull overestimating the instantaneous volume (and its fluctuations) whenever sparse protrusions are present, which in turn biases the inferred compressibility. These convex-hull-based results

therefore provide a useful cross-check, while also highlighting why the surface mesh method is adopted as the reference characterization in the main text.

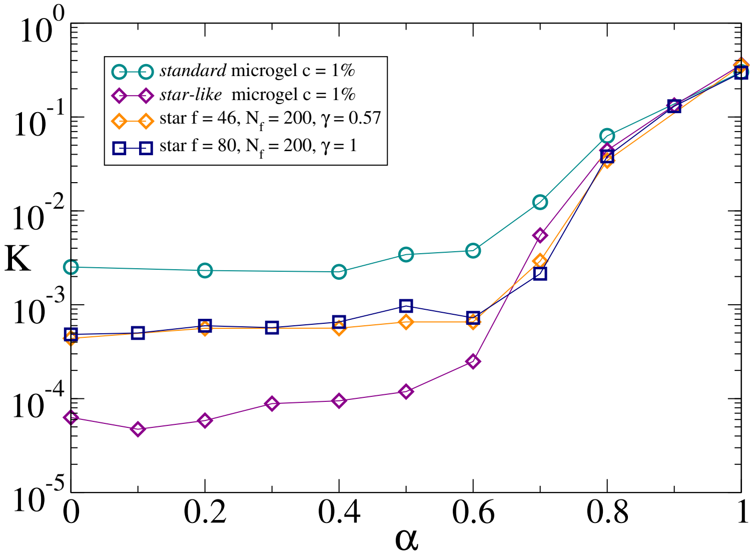

Figure S8: Bulk modulus K in units of $k_{\mathrm{B}}T/\sigma^3$ as a function of effective temperature α, obtained using the convex hull method to estimate the volume. Note that this method yields systematically smaller values of K for the *star-like* microgel, due to its sensitivity to occasional long, sparse chains.
